# GRADE Good Practice Statements – A time to say “Good-bye”? A new typology for normative statements on interventions

**DOI:** 10.1016/j.jclinepi.2024.111371

**Published:** 2024-04-25

**Authors:** Susan L. Norris

**Keywords:** GRADE, good practice statements, guidelines, recommendations, public health

## Abstract

**Background:**

Clinical and public health guidelines include a variety of types of normative statements concerning interventions. “Recommendations” are usually the central focus, and are based on one or more systematic reviews of research evidence. Guidelines may include other types of normative statements, however, including GRADE good (or best) practice statements (GPS), which represent recommendations that guideline panels feel are important but are not appropriate for formal ratings of quality of evidence because it is sufficiently obvious that desirable effects outweigh undesirable effects. These normative statements are typically supported by a great deal of high-certainty, indirect evidence, which the authors feel would be a waste of time to examine.

There are a number of conceptual and methodological issues with GRADE GPS, however, and these are manifest in guidelines, including both inappropriate over- and under-use, and unclear interpretation and impact among end-users. This situation has arisen in part from lack of clarity in, and misunderstandings of, GRADE guidance; the lumping of many different types of normative statements under one label (“GPS”); from limitations in GRADE’s approach to linked bodies of evidence; and because the appropriate basis for many normative statements about interventions is not reviews of research evidence. A new typology is needed for normative statements on interventions and policies that are not optimally based on reviews of research evidence.

**Proposed typology:**

This proposed typology differentiates normative statements about interventions by the type or nature of the most appropriate basis for the statement. The typology encompasses the range of statements encompassed by GPS, but provides a more nuanced categorization designed to assist both guideline developers and end-users.

This typology encompasses two main types of normative statements about interventions (including policies): 1) statements that indicate when to use (or not) an intervention, which intervention to use, and if, when and how to use it; and 2) the principles, practices or norms that inform or underpin such interventions. These correspond to normative statements based on empirical evidence, and those based on human rights, ethics or norms, respectively.

Normative statements based on empirical evidence include: 1) recommendations based on systematic reviews of human or animal evidence on effectiveness and harms, including linked bodies of evidence; 2) normative statements based on scientific fundamentals (e.g., physical/biological/chemical properties, theories, laws, or principles); and implementation guidance based most commonly on experiential evidence such as case studies.

Normative statements based on human rights, ethics or norms include: 1) guiding principles, based on human rights standards and conventions and/or ethics principles; and 2) practice norms and standards, based on clinical and public health norms and/or professional standards.

**Conclusions:**

There are conceptual and methodological problems with GRADE GPS, leading to their misapplication and over- and under-use. This paper presents a proposal for a new typology for normative statements on interventions, according to the basis for the statement. This typology encompasses and replaces GPS, providing a more nuanced set of statements. Testing of this proposed approach is needed among both guideline developers and end-users.

## 1 Introduction

Clinical and public health guidelines include a variety of types of normative statements concerning interventions. Normative statements tell the reader what do to or how to do an action, in contrast to descriptive statements. “Recommendations” are usually the main type of normative statement in guidelines, and are based on one or more systematic reviews of research evidence and developed using the GRADE approach to assessing certainty of the body of evidence and a structured process for formulating recommendations [1]. Guidelines may include other types of normative statements, including good (or best) practice statements (GPS) [2–4].

The GRADE Working Group states that GPS “represent recommendations that guideline panels feel are important but that, in the judgment of the … GRADE working group are not appropriate for formal ratings of quality of evidence” [3] because it is sufficiently obvious that desirable effects outweigh undesirable effects such that no direct evidence is available because no one would conduct a study to examine the issue. These normative statements typically are supported by a great deal of high-certainty, indirect evidence, which the authors note would be “challenging and a waste of time and energy” to examine [2, 5]. The authors also note that “One way of recognizing such questions is that if one made the alternative explicit, it would be bizarre or laughable” [2] or the unstated alternative clearly does not conform to ethical norms [3]. According to these authors, guideline panels can mistakenly try to collect direct evidence, which will be sparce, hence generating low or very low certainty evidence and potentially a conditional recommendation, when, in fact, relevant indirect evidence is unequivocal [5]. They warn, however, that GPS should be used sparingly due to their subjective nature [3].

More recently, GRADE Working Group members [6] proposed additional guidance on how to identify the need for GPS, and a structured process for their development and reporting. They note that practical considerations such as guideline timeline and available resources can help determine the type of normative statement. As well, direct evidence or the lack of a compelling body of linked, indirect evidence should lead to issuance of a recommendation with an accompanying GRADE assessment of certainty of evidence [6].

## 2 Problems with the GRADE approach to Good Practice Statements

There are a number of conceptual and methodological issues with GRADE GPS.

First, the GRADE approach focuses on systematic reviews of research evidence (usually human) when assessing the benefits and harms of clinical and public health interventions. GRADE does not explicitly acknowledge that many normative statements about interventions are not optimally based on such evidence, or at least not the type identified in bibliographic databases traditionally examined in systematic reviews. In fact, some normative statements about interventions are appropriately not based on research evidence or empirical data at all: for example, “All women have the right to evidence-based, comprehensive contraceptive information, education and counselling to ensure informed choice.” [7].

Second, the GRADE approach is predicated on using a single body of direct evidence as the basis for recommendations, usually assuming a one-to-one relationship between the PICO (population, intervention, comparator, outcome) (systematic review) question and the “recommendation question”. Thus, when several linked bodies of evidence are required to answer a recommendation question (each body derived from a PICO question) and the expert panel is certain of the net benefits (despite no systematic review of the individual PICO questions for each link in the evidence chain), GRADE suggests formulating a GPS which is equated with a strong recommendation [6]. GRADE does not provide explicit methods for assessing certainty of evidence across multiple bodies of linked evidence (except in the context of network meta-analysis [8] and for diagnostic tests [9]), considering them as “indirect” and therefore low or very low certainty. Thus, when multiple bodies of linked evidence are needed to inform a normative statement about an intervention, this will usually support only a conditional recommendation, even when each individual body of evidence is high certainty. Such a simplistic paradigm is often unhelpful for public health recommendations about interventions, which may require examination of multiple bodies of evidence addressing multiple research (PICO) questions, best depicted via an analytic framework or similar conceptual model [10, 11].

Third, official GRADE guidance on GPS is unclear and difficult to operationalize. This guidance does not clarify what constitutes evidence that is “not appropriate” for certainty of evidence assessment [5]. In addition, two of the five criteria for GPS (2016) [3] are subjective: that implementation is likely to result in large net positive consequences (given that the body of evidence has not been formally reviewed); and that the identification and summarization of evidence would be a “poor use of guideline panel’s limited time and energy”. Two other criteria are not specific to GPS: that the message is necessary and there is a well-documented rationale. Finally, the older criterion of a comparator that is “laughable” [2] is highly subjective, can be context- and culturally-specific, and is not always the case when GPS are formulated based on indirect or linked evidence (e.g., where the intervention is screening for a disease or condition).

Fourth, although not official GRADE guidance, GRADE Working Group members indicate that the availability of certain types of evidence, the guideline timeline, and available resources may affect whether a GPS or “GRADEd” recommendation is formulated [6]. These factors should *never* determine the type of normative statement – rather the nature of the intervention or question should drive the determination of the optimal type of data, information, evidence, or other inputs that inform the normative statement, and its type.

Finally, GPS as defined in GRADE encompass a broad range of types of statements with different bases, from specific guidance on implementation (potentially based on experiential evidence), to statements about patients’ rights (based on human rights conventions). Some of these statements do not logically fit the label of “Good Practice Statement”, yet GRADE lumps them all together, which may prove challenging for interpretation by end-users. For example, while a statement such as “Clean the skin with antiseptic prior to giving an injection” might be deemed “good practice”, it is illogical to label normative statements such as “Patients have the right to access affordable and high-quality health care” and “Do not jump out of an airplane without a parachute” as “good practice”.

## 3 Demonstrated problems with Good Practice Statements in guidelines

These conceptual and methodological problems with GRADE GPS are manifest as problems in clinical and public health guidelines, including both over- and under-use of GPS [12, 13].

First, expert panels may formulate GPS when they should rather have crafted a GRADEd recommendation. This can arise when the panel examines a body of direct evidence (using standard methods) and finds it insufficient (including low- or very low-certainty evidence) to make the strong recommendation they feel is appropriate. The panel may then formulate a GPS in favor of the intervention despite the lack of evidence, due to preconceived opinions or biases. This is a miss-application of the GRADE approach: rather, “GRADEd” recommendations should be issued.

One reason the systematic review may have identified a low- or very low-quality body of evidence is that the review team looked only for *direct* evidence on health or other outcomes that were deemed critical or important. For many interventions, for example screening, such “overarching” evidence may not be available, and the more appropriate approach would have been to seek evidence on several key (“PICO”) questions that need to be answered to address the recommendation question “Should screening intervention X be used in population Y?”. As noted above, GRADE does not have explicit methods for assessing linked bodies of evidence, and thus methodologists conclude the evidence is “indirect” and therefore low- or very-low certainty regardless of the quality of the bodies of evidence used to inform each individual key question. Expert groups that feel a strong recommendation is indicated may thus make a GPS.

Second, guideline developers may formulate recommendations based on a systematic review and GRADE assessment of certainty of evidence, when, in fact, a GPS is indicated per current GRADE guidance [13, 14]. This is well documented among WHO guidelines, wherein 18% of discordant recommendations (strong recommendations based on low or very-low quality evidence) should have been presented as a GPS [13, 14].

This situation may occur when a systematic review of human research evidence is not the optimal basis for the recommendation, yet the guideline developers proceeded with such, trying to apply GRADE methods. Other types of empirical data and evidence appropriately inform many normative statements. Examples include drug dosing and contraindications based on pharmacokinetic data, or using experiential evidence to formulate “how to” implementation guidance where high certainty is not needed when providing practical management options.

Finally, there is little known about expert guideline panels’ or guideline end-users’ perceptions of GPS, and how they view them in comparison to a strong recommendation.

In summary, GRADE GPS are problematic for guideline developers with both under- and over-use, and unclear interpretation and impact among end-users. This situation may have arisen in part from misunderstandings of GRADE guidance, in part because GRADE guidance is unclear, in part because of limitations in GRADE’s approach to linked bodies of evidence, and because many normative statements about interventions are appropriately based on inputs other than reviews of research evidence. A new typology is needed for normative statements that are not optimally based on reviews of research evidence.

## 4 Development of a new typology for normative statements

The proposed new typology for normative statements was developed via an iterative process and examination of a large cohort of guidelines. Development of a framework for types of normative statements issued by the World Health Organization (WHO) began with an informal evaluation of all WHO guidelines published between 2008 and 2016 that included a normative statement labelled a GPS or similar (e.g., “best practice statement”, “guiding principle”, etc.). This work was continued with an assessment of WHO guidelines published 2022-2023, with subsequent evolution of the typology to its current form. I iteratively applied the typology to items in the guideline cohort, revising and refining the typology to its current form as I assessed whether each subsequent statement reasonably fit the typology. In addition, I searched PubMed on 7 March 2023 using the search strategy: ((“good practice statement$”[Title/Abstract]) OR (“best practice statement$”[Title/Abstract])) Filters: in the last 10 years, English). Relevant articles were reviewed and examples of GPS within those papers were examined for their “fit” into the proposed typology.

## 5 Proposed typology for normative statements

This proposed new typology for normative statements regarding interventions aims to address the problems outlined above. I propose that such normative statements are differentiated by the type or nature of the basis for the statement. This typology encompasses the range of statements encompassed by GRADE GPS [3], but provides a more nuanced categorization designed to assist both guideline developers and end-users, by helping to ensure that normative statements have the appropriate basis and are optimally presented.

This typology encompasses two main types of normative statements about interventions (including policies): i) statements that indicate when to use (or not) an intervention, which intervention to use, and if, when and how to use it; and ii) the principles that inform or underpin such interventions. These correspond to normative statements based on empirical evidence, and those based on human rights, ethics or norms, respectively.

The proposed types of normative statements are:

- Normative statements based on empirical evidence
  1. **Recommendations** based on systematic reviews of human or animal evidence on effectiveness and harms, including linked bodies of evidence
  2. **Normative statements based on scientific fundamentals** (e.g., physical/biological/chemical properties, theories, laws, or principles)
  3. **Implementation guidance** based most commonly on experiential evidence such as case studies
- Normative statements based on human rights, ethics or norms
  4. **Guiding principles**, based on human rights standards and conventions and/or ethics principles
  5. **Practice norms and standards**, based clinical and public health norms and/or professional standards

Each of the five types of normative statements is described in more detail below; Table 1 provides a summary of each type and examples.

### 1 Recommendations

The type of normative statement that guideline developers are most familiar with is “recommendations”, which are based on empirical evidence identified and summarized via a systematic review of research evidence (human or animal) on effectiveness and adverse effects at a minimum, and accompanied by an assessment of the certainty of the body of evidence. Structured consideration of the evidence and other factors leads to a “strong” or “conditional” recommendation, per GRADE guidance [1].

Any recommendation question about an intervention that should optimally be based on a review of human or animal research evidence is labelled a “recommendation” and an assessment of the certainty of evidence should be provided. Such normative statements should *never* be reformulated as any other kind of normative statement after a review of the evidence reveals little or no direct evidence. When direct evidence is sparse or when indirect evidence is used, the certainty of evidence will likely be low or very low, and the recommendation will usually be conditional. For example, early in the COVID-19 pandemic in 2020 when there was no direct evidence on SARS-CoV2, its transmission or resultant illness, the normative statements issued by guideline developers were appropriately called “recommendations” (and not GPS) as they were based on all known direct and indirect (from related viruses) human research evidence.

Some recommendation questions are best addressed with multiple bodies of evidence, linked across a causal pathway (as depicted in an analytic framework or other conceptual model). Each body of evidence should be assessed using GRADE, and a single assessment then provided across the linked evidence. While GRADE has not provided guidance on this (except for network meta-analysis [8] and diagnostic test assessments [9]), the US Preventive Services Task Force has explicit and effective methods for assessing the quality of a body of linked evidence for screening interventions [15].

### 2 Normative statements based on scientific fundamentals

Normative statements based on scientific fundamentals are also based on empirical data; however, such data are not optimally synthesized with a systematic review of human or animal research evidence. Such data are usually noncomparative, and may or may not be experimental. They are often related to the properties of the intervention or its components, and are frequently laboratory-based. Examples include recommendations on drug dosing or drug-drug interactions which are based on pharmacokinetic and other biochemical properties, or the selection of masks to prevent disease transmission based on size of the pathogen and filter properties of the mask material. The facetious example of a recommendation to not jump out of a flying airplane without a parachute is based on the law of gravity, combined with physical principles of mass effects and friction, among others [16].

### 3 Implementation guidance

Implementation guidance refers to normative statements that tell the reader how to implement a recommended intervention. Such guidance should be linked to the “mother” recommendation, and may be focused on the individual patient, procedure or other action, or on the healthcare or other system. Implementation guidance is often quite granular, with specific details such as optimal needle size or anatomic location of medication injection. On the other hand, implementation guidance can also apply at the health system or program level, such as when and how to collect data for a disease registry, or guidance on how to achieve efficient patient flow in a healthcare setting.

Implementation guidance is commonly based on experiential evidence including case studies or case series, when there are one or more reasonable options for implementation. When there is significant uncertainty regarding the optimal approach, then a systematic review of effectiveness evidence may be indicated and a recommendation formulated.

### 4 Guiding principles

Guiding principles are a type of normative statement that is based on human rights standards and conventions, or on ethics principles. In contrast to the first three types of normative statements, these are not based on empirical data or evidence.

Human rights are norms and standards that are understood to be inherent and inalienable to all human beings; these have in many countries been codified into law and practice. Some of these codified norms exist across international boundaries as international conventions (e.g., the International Covenant on Social, Cultural and Economic Rights [17]) and treaties, while others have been incorporated into regional or national instruments. These standards are underpinned by specific principles including equality and non-discrimination which require that no one should face discrimination on the grounds of any one of a set of specific characteristics including race, gender, ethnicity and religion.

Guiding principles based on human rights norms and standards can provide a legal basis for health-related laws and policies (e.g., access to safe abortion) or enable legal environments for key populations at higher risk of disease (e.g., protections against discrimination and the right to participation for people living with HIV or people who use drugs).

There are four main principles of clinical ethics: beneficence (the obligation of healthcare workers to act for the benefit of the patient or population), nonmaleficence (the obligation of healthcare workers to not cause harm), autonomy (all persons have intrinsic and unconditional worth, and thus should have the power to exercise self-determination), and justice (fair, equitable, and appropriate treatment and distribution of resources) [18, 19].

For public health, there are additional principles [20]. *Health maximization*, refers to the obligation of public health to maximize health in the populations they serve. The principle of *efficiency* in public health is important, given that there is always more need than available resources [20]. Thus, there is a moral duty to use scarce health resources efficiently. Such an approach enables public health workers to produce maximize health benefits for greatest numbers of people.

Finally, the principle of *proportionality* refers to balancing individual freedom against wider social goods, and that considerations will be made in a proportionate way [20]. Additional ethics principles are proposed in public health, including the precautionary principle and principles of solidarity or social cohesion [21].

Both clinical and public health principles are underpinned by morality: both *common morality and particular morality. Common morality* is the most relevant to guiding principles, and encompasses the moral norms of conduct that transcend group characteristics, social norms, geography, religion, etc. Common morality includes mandates not to kill or harm others, not to steal, not to punish the innocent, to obey the law, and to assist persons who are suffering, as examples. On the other hand, *particular morality* refers to norms that bind groups because of their culture, religion, or profession and includes responsibilities, ideals, and professional standards, among others [18].

In clinical medicine and in public health, there can be conflicts among these principles as applied to a specific situation. At times the principle of health maximization may come into conflict with the principles of beneficence and non-maleficence [20]. Beneficence and autonomy can be at odds when a healthcare worker recommends a specific treatment, but a competent patient refuses.

Guiding principles are invariably rather general statements. They apply across a range of interventions and recommendations, and are often presented early in a guideline as they underpin subsequent recommendations and other normative statements.

### 5 Practice norms and standards

The fifth type of normative statement is based on formal and informal norms [18], and/or on particular morality (defined above), as they relate to professional responsibilities, ideals and standards in the context of the clinical and public health systems. Formal norms are codified, such as in professional codes of practice or conduct, whereas informal norms are based on accepted practice. Within the specified context, there is high certainty of benefit, as well as acceptance from a health system or public health perspective, without interfering with human rights or ethics principles.

Practice norms and standards are usually presented as a more general statement, in contrast to specific guidance which is more suitable for either recommendations (where there is significant uncertainty as to what to do and a systematic review is needed), or implementation guidance (where some uncertainty may be acceptable and one or more options may be appropriate). Examples of practice norms and standards include statements that patients should be followed up post-procedure; that monitoring is needed for adverse drug effects; that health services should have mechanisms in place for clinical governance, and for monitoring and evaluation; or that malnourished children should be tracked during management.

## 6 Considerations when implementing the new typology

There are a number of potential considerations when formulating any type of normative statement, and when using the proposed new typology. First and foremost, if during the scoping and planning stage, the developers decide that the key questions underpinning a normative statement should be based on a systematic review of human (or animal) research evidence, then the normative statement is a *recommendation*, and the expert panel should never transform the statement into another type. Formulating a GPS in these situations is a miss-application of GRADE and formulating something other than a recommendation is a miss-application of the proposed new typology. This includes the situation where the evidence is insufficient to make a strong recommendation yet the expert guideline panel considers such to be justified. As well, this includes the situation where indirect evidence or multiple linked bodies of evidence are needed to address the recommendation question or when no direct (overarching) evidence was identified, yet the expert panel believes the benefits outweigh the harms.

When a recommendation question requires exploration of multiple key questions using systematic reviews of research evidence on effectiveness and harms, a GRADE assessment of certainty of evidence should be made for each body of evidence. This should be followed by an assessment of certainty in aggregate across the multiple bodies of evidence along the causal pathway. Although GRADE currently does not have methods for this situation, a well-developed approach is available [15].

For each normative statement, there is usually one primary consideration or basis, which is the focus of this proposed typology. For recommendations, the primary basis is usually a systematic review of research evidence (usually human) on benefits and harms of the intervention. For a guiding principle the primary basis is an ethics principle or human rights standard. However, other considerations often come into play when normative statements are formulated. For recommendations, these other considerations are usually enumerated in an evidence-to-decision (EtD) framework (whether GRADE [22], WHO-INTEGRATE [23], or another). Each EtD consideration is, in turn, based on specific inputs, whether empirical evidence (including systematic reviews of research evidence), human rights, ethics principles, expert opinion or costs, etc. These “secondary” considerations (and their respective bases), do not define the type of normative statement, however they are usually outlined in the rationale statement accompanying each normative statement. For example, the human right to accessible, quality healthcare might, in part, underpin a recommendation for a screening intervention which is primarily based on evidence on diagnostic accuracy and net health benefits. Nonetheless, this normative statement will be classified as a “recommendation”, given that its primary basis is a systematic review of the empirical evidence of health benefits of the screening test.

Some normative statements are based on several different types of empirical evidence. For example, the use of masks in the community might involve examination of pathogen characteristics and mechanisms of transmission, mask filter size and fit, compliance with mask use, physical distance between individuals, and indoor ventilation and air flow, among others. Some of these questions are best addressed with laboratory studies and physical properties, while others may rely on qualitative or quantitative human research evidence. Thus, it may be challenging to label the normative statement as a single type. However, labelling is much less important than ensuring that the optimal inputs were used to inform or support the normative statement. The type of normative statement should be based on the most relevant or important inputs, while clarifying in the rationale statement the various bases for the statement.

Multi-component normative statements that combine a statement based on empirical evidence with one primarily based on human rights and/or ethics principles or norms should generally be avoided. For example, combining a guiding principle with a recommendation may obfuscate the basis for each part of the normative statement. Similarly, recommendations based on a systematic review of human research evidence should be presented separately from normative statements based primarily on other types of empirical data.

The basis for every normative statement and the outcomes considered or assessed must be explicit and transparent to the reader. For example, there may be major differences between findings in a laboratory on the transmission of viruses through masks, and the effectiveness of masks when used at the community level to reduce disease incidence. However, this does not mean that one should never make a recommendation based on laboratory data, but rather that caution is needed when making normative statements based on intermediate or surrogate outcomes, and the basis for all statements must be made explicit to the reader.

This proposed typology requires extensive review and testing prior to widespread application by guideline developers. Testing could examine: 1) comprehension (are the categories understandable and implementable?); 2) reliability (Kappa statistic among independent assessors for selected cohorts of normative statements); and, most importantly, 3) testing of face and content validity, and comprehensiveness across a broad range of clinical and public health normative statements that are currently classified as either “recommendations” or “GPS”. Through testing of a large number of existing normative statements, it will become apparent if the typology is comprehensive, or if simplification might be indicated.

Finally, the introduction of any new perspective, framework or paradigm may engender confusion among guideline developers and end-users. Once tested and the next iteration finalized, key entities that develop guidelines will need to familiarize themselves with the finalized framework, understand its relationship to GRADE GPS (it replaces them), and have the opportunity to provide constructive feedback and dialogue as experience with it is gained and evaluations are published.

## 7 Conclusion

Current methods guidance on normative statements other than recommendations, including GRADE GPS, is inadequate and frequently misunderstood. This has led to the misapplication and over- and under-use of GPS. I propose a new typology for normative statements on interventions, according to the nature of the data, evidence or other basis for the statement. This typology encompasses and replaces GRADE GPS, providing a more nuanced set of types of statements with the goal of increased transparency, comprehension, and impact. Testing among guideline developers should address the usability and comprehensiveness of this proposed new approach, and among end-users, focus on understanding and impact.

## Figures and Tables

**Table 1:** Types of normative statements about interventions or policies

Type of statement	Definition	Basis/input(s)*	Examples
**Based on empirical data or evidence**			
1. Recommenda tion	A normative statement about an intervention that: 1) tells the intended end-user what they can or should do in specific situations to achieve the best possible health or other relevant outcomes, individually or collectively; and 2) addresses an area of uncertainty that should optimally be answered with a systematic review of research evidence on benefits and harms, at a minimum.	Empirical research evidence (human or animal) on benefits and harms: direct and/or indirect evidence (including multiple linked bodies of evidence) identified and synthesized as a systematic (including rapid) review. Comment: Other types of inputs can contribute to decision-making for some evidence-to-decision considerations (e.g., human rights, expert opinion, cost data, etc.).	“WHO suggests that non-sugar sweeteners not be used as a means of achieving weight control or reducing the risk of noncommunicable diseases (*conditional recommendation, low certainty evidence*)” [24] “The use of HCV [hepatitis C virus] point-of-care (POC) RNA NAT [ribonucleic acid nucleic acid testing] assays can be an alternative approach to laboratory-based HCV RNA NAT assays to diagnose HCV viraemic infection. (low/moderate certainty evidence” [25]
2. Normative statement based on scientific fundamentals	A normative statement about an intervention that: 1) tells the intended end-user what they can or should do in specific situations to achieve the best possible health or other relevant outcomes, individually or collectively; and 2) is based on scientific fundamentals, including scientific theory, laws, principles, facts, or hypotheses.	Mechanistic or laboratory-based empirical evidence; scientific theories; physical, chemical biological principles, properties or laws	The final concentrations for handrub formulations are: “ethanol 80%…, glycerol 1.45%…, hydrogen peroxide 0.125%…” [26]
3. Implementati on guidance	A normative statement that provides specific guidance on when, how and/or where to implement a recommended intervention. Guidance may be aimed at individuals, populations, or health systems.	Experiential evidence, expert opinion, case studies, case series Comment: Where there is significant uncertainty, a systematic review may be performed and a recommendation formulated.	“As part of efforts to implement these recommendations, health system stakeholders should consider the need to ensure: one scan before 24 weeks of gestation for accurate estimation of gestational age…” [27]
**Based on human rights, ethics, or norms**			
4. Guiding principle	A high-level normative statement that provides guidance or principles that underpin a recommendation or other normative statement, based on human rights standards or conventions, or ethics principles.	Human rights conventions and standards, or ethics principles	“Countries should work towards implementing and enforcing antidiscrimination and protective laws, derived from human rights standards, to eliminate stigma, discrimination and violence against people from key populations” [28] “Health services should be made available, accessible and acceptable to people from key populations, based on the principles of medical ethics, avoidance of stigma, non- discrimination and the right to health.” [28]
5. Practice norms and standards	A normative statement of standards of professional conduct, responsibilities, ideals or standards, based on accepted formal or informal norms and/or standards in a specific healthcare context. These norms must not be contrary to human rights conventions or ethics principles.	Formal (codified) or informal (based on accepted practice) norms as they relate to professional conduct, responsibilities, ideals and standards in the context of the clinical and public health systems. Comment: These normative statements may also have a basis in ethics principles, thus there may be overlap with Guiding Principles. This type of statement is more general than implementation guidance.	“Both microscopy and RDTs [rapid diagnostic test] should be supported by a quality assurance programme.” [29] “In areas with ongoing local malaria transmission…, vector control interventions should not be scaled back. Ensuring access to effective malaria vector control at optimal levels for all inhabitants of such areas should be pursued and maintained.” [29] “Decisions about whether an infant less than 6 months of age at risk of poor growth and development needs a supplementary milk in addition to breastfeeding must be based on a comprehensive assessment of the medical and nutritional/feeding needs of the infant, as well as the physical and mental health of the mother/caregiver.” [21]

* Some normative statements are based on more than one “input”. The type of normative statement is based on the most relevant or important inputs, while other evidence-to-decision considerations may also be important when formulating the statement.

## What is new?

GRADE good practice statements (GPS) are problematic with respect to their conceptualization and guidance.

This paper proposes a new typology for normative statements about interventions and policies, based on the optimal basis for the a given statement. This typology encompasses and replaces GPS, providing a more nuanced set of statements to facilitate developers’ understanding and methods, and end-users’ comprehension and impact.

Testing of this proposed new approach is needed among both guideline developers and end-users.
